# Supplementary material for: Automated inference of disease mechanisms in patient-hiPSC-derived neuronal networks
Source: Commun Biol. 2025 May 20;8:768. doi: 10.1038/s42003-025-08209-2 (PMC12092834; doi:10.1038/s42003-025-08209-2)
Supplement: Supplementary file 2 — Supplementary Information [file 42003_2025_8209_MOESM2_ESM.pdf]

Supplemental Information

Automated inference of disease mechanisms in patient-hiPSC-derived neuronal networks

Nina Doorn[1,*], Michel J.A.M. van Putten[1,2], and Monica Frega[1,3]

[1]Department of Clinical Neurophysiology, University of Twente, 7522 NB Enschede, The Netherlands
[2]Department of Neurology and Clinical Neurophysiology, Medisch Spectrum Twente, 7512 KZ Enschede, The Netherlands
[3]Department of Informatics, Bioengineering, Robotics and Systems Engineering, University of Genoa, 16145 Genoa, Italy
[*]Correspondence: n.doorn-1@utwente.nl

Supplementary Methods

Embedding Network for Data-Driven Summary Statistics

To assess whether data-driven summary statistics could improve parameter inference, we implemented a convolutional embedding network trained jointly with the neural density estimator (NDE). Our goal was to explore whether such embeddings could outperform our hand-crafted MEA features.

We generated 100,000 simulations with parameter configurations sampled from our box prior. Input to the embedding network consisted of multi-unit firing rates binned at 100 ms over a 3-minute recording, resulting in input tensors of size 12 (electrodes) $\times$ 1800 (time bins).

The embedding network was designed to extract both local spatio-temporal features and long-range temporal dependencies. Its architecture included:

1. A 2D convolutional layer (kernel size 3×5) to capture local electrode-time patterns;
2. A depthwise convolution (1×5 kernel) to further model temporal structure;
3. A 1×1 convolution for integrating information across electrodes;
4. Two dilated convolutions (1×5 kernel, dilation rates 2 and 4) to capture long-range dependencies;
5. Two max-pooling layers (1×2 kernel) for dimensionality reduction;
6. A flattening layer, followed by a fully connected ReLU layer (256 units), and a final linear projection to a 20-dimensional embedding space.

We trained this embedding network jointly with the NDE using the Python package sbi, version 0.23.0. For comparison, we also trained a separate NDE using the hand-crafted MEA summary statistics extracted from the same simulations. Due to the complexity of the embedding network and the high dimensionality of the input, training and inference were approximately one hundred times slower than when using hand-crafted features. This, together with limitations in computational resources, prevented extensive architecture tuning.

Supplementary Figures

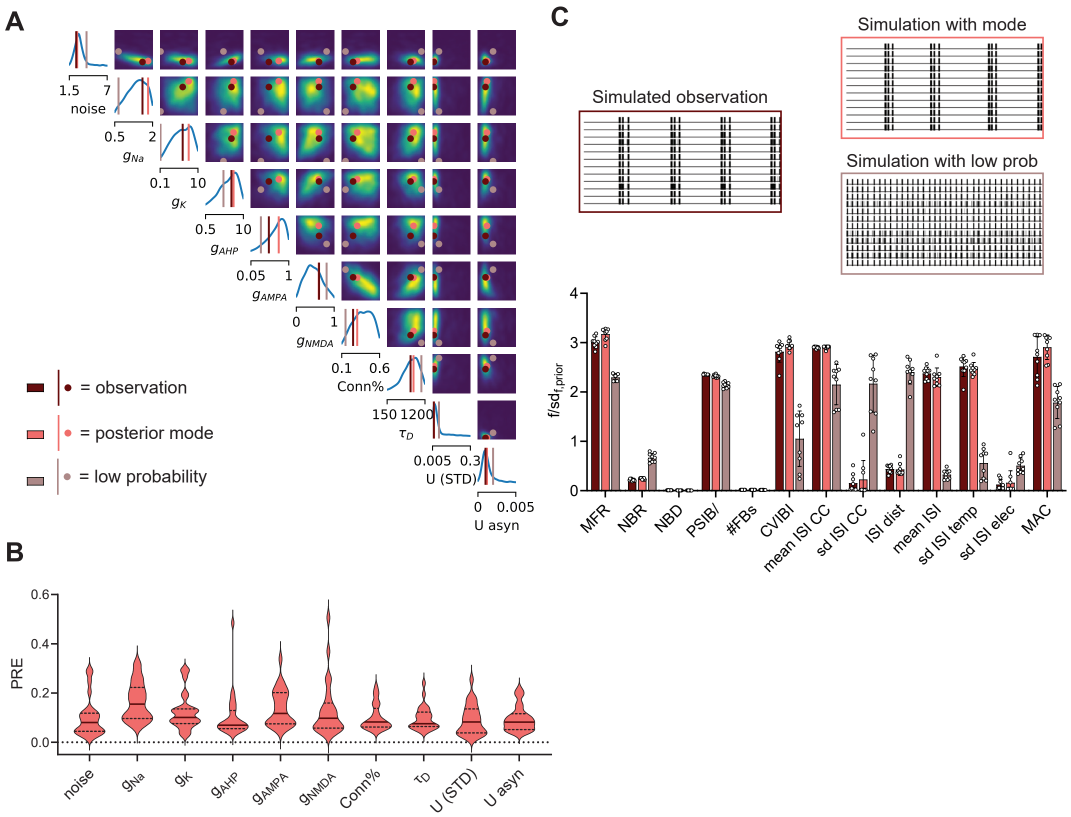

Figure S1: **Posterior predictive check shows SBI can correctly identify ground-truth parameters. A)** Inferred posterior for 10 model parameters given 13 MEA features of simulated activity. Ground-truth model parameters are shown in brown, posterior mode in pink, and low-probability model parameters in beige. **B)** Violin plots for Parameter Recovery Error (PRE) values, as defined by[1], for each parameter in each of 25 posterior-predictive checks with different ground-truth parameter sets. **C)** Top: Raster plots showing 1 minute of (left) simulation used as input for the inference, (top right) example simulation with the mode of the posterior distribution, and (bottom right) example simulation with the low probability model parameters. Bottom: MEA features of simulations ($n = 10$ per condition) with the ground-truth model parameters (brown), the mode of the posterior (pink) and low probability model parameters (beige). The MEA features are: mean firing rate (MFR), network burst (NB) rate (NBR), NB duration (NBD), percentage of spikes in NBs (PSIB), the number of fragments per NB (#FBs), the coefficient of variation of the inter-burst-intervals ($\mathrm{CV_{IBI}}$), the average correlation coefficient between channel signals (mean CC), the standard deviation (sd) of the CCs (sd CC), the inter-spike interval (ISI) distance (ISI dist), the mean ISI, the sd of ISIs over time (sd ISI temp), the sd of ISIs between electrodes (sd ISI elec) and the maximum autocorrelaction component (MAC). Data shows mean ± sd.

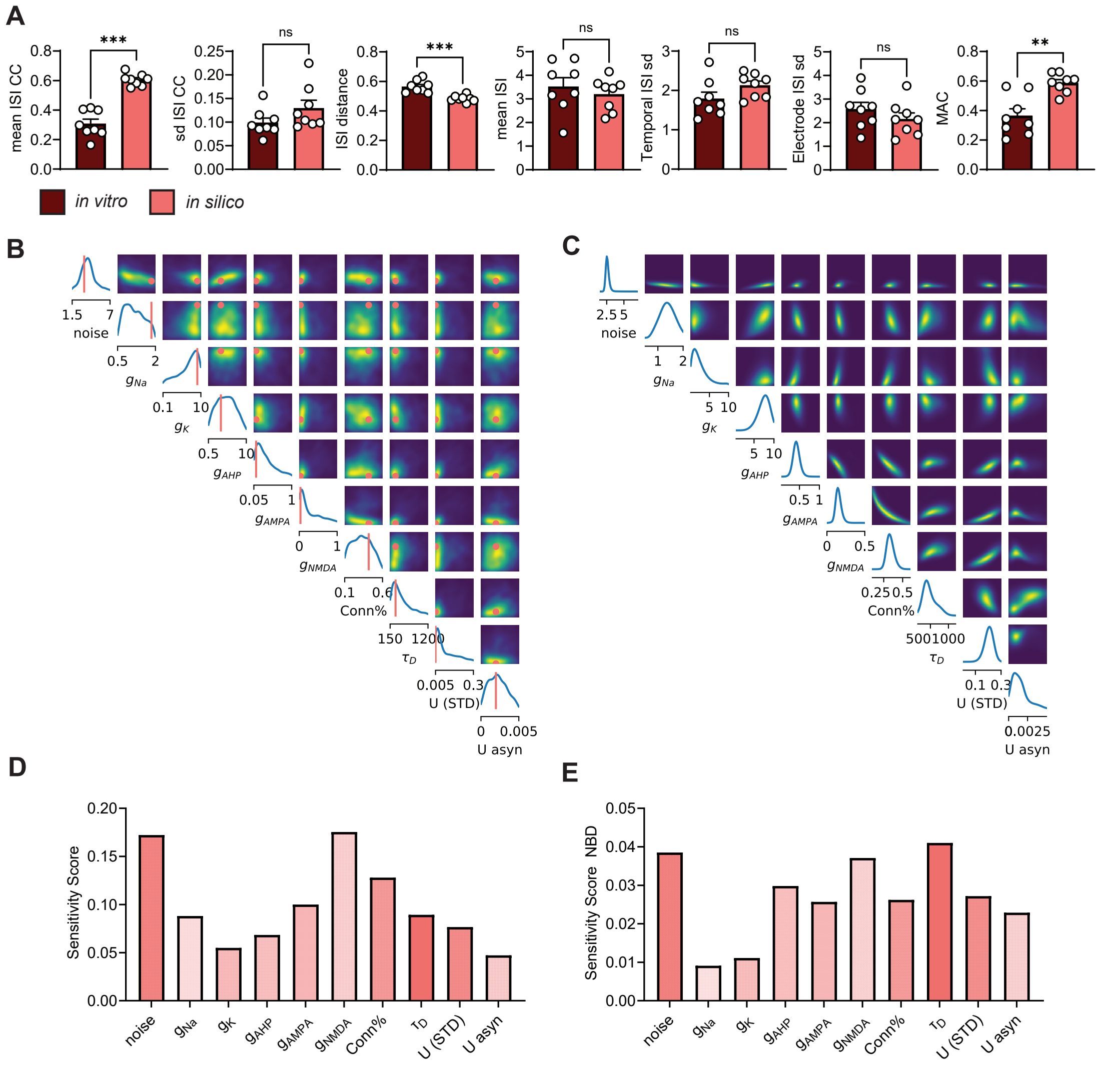

Figure S2: **SBI can correctly identify constrained parameters resulting in simulations mimicking healthy neuronal network behavior and their corresponding sensitivities.** **A)** MEA features of *in vitro* measurements and simulations with the mode of the posterior (pink). The features are: mean firing rate (MFR), network burst (NB) rate (NBR), NB duration (NBD), percentage of spikes in NBs (PSIB), the number of fragments per NB (#FBs), the coefficient of variation of the inter-burst-intervals (CV_{IBI}), the average correlation coefficient between channel signals (mean CC), the standard deviation (sd) of the CCs (sd CC), the inter-spike interval (ISI) distance (ISI dist), the mean ISI, the sd of ISIs over time (sd ISI temp), the sd of ISIs between electrodes (sd ISI elec) and the maximum autocorrelaction component (MAC). Bars show mean $\pm$ SEM. **B)** Inferred posterior distribution of healthy neuronal networks of a different MEA batch compared to Fig. 2B. **C** Complete conditional distribution of the subset shown in Fig. **??**C). Plots on the diagonal show conditional distribution when all other parameters are fixed. For off-diagonal plots we keep all but two parameters fixed. **D)** Sensitivity scores computed using eigendecomposition of the posterior shown in Fig. 2B). **E)** Sensitivity scores of the posterior computed with a neural network trained to predict the Network Burst Duration (NBD) feature from the parameters. Scores indicate how much the NBD feature is influenced by the model parameters.

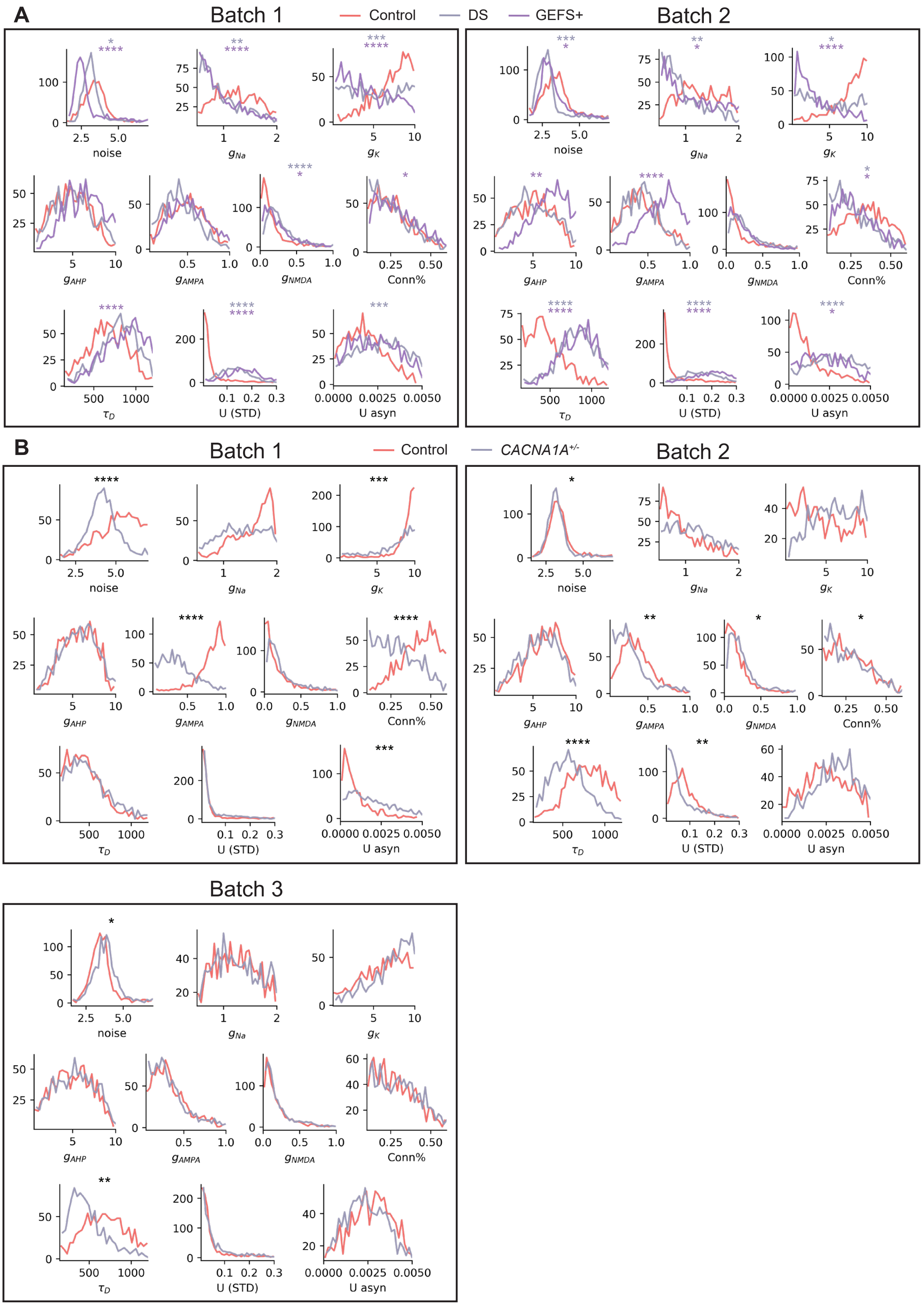

Figure S3: **Comparisons between univariate marginals of healthy and diseased neuronal networks from different batches. A)** Comparisons of two batches between neuronal networks of a healthy control, a patient with DS and a patient with GEFS+. **B)** Comparisons of three batches between neuronal networks of a healthy control and *CACNA1A* deficient networks. Marginals were compared using a Kolmogorov-Smirnov (KS) test test with 50 samples per marginal. * p <0.05, ** p <0.01, *** p <0.001, **** p <0.0001.

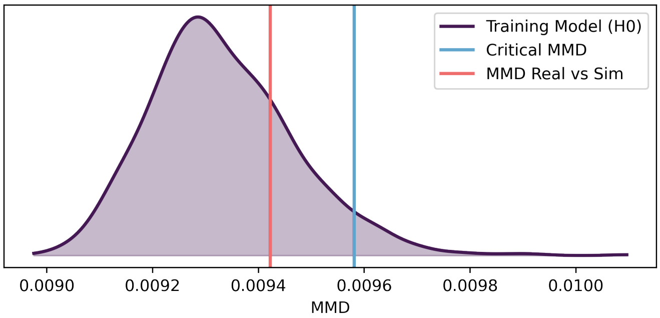

Figure S4: **Check for model misspecification** using Maximum Mean Discrepancy (MMD) as proposed by Schmitt et al.[2]. Shown is the MMD sampling distribution under the training model (H0), the critical MMD value based on its 95-percentile, and the MMD of all experimental data used (MMD Real vs. Sim). Summary features were normalized and whitened. MMD was computed using an inverse multiquadratic kernel with h=0.1 as suggested by Ardizzone et al.[3].

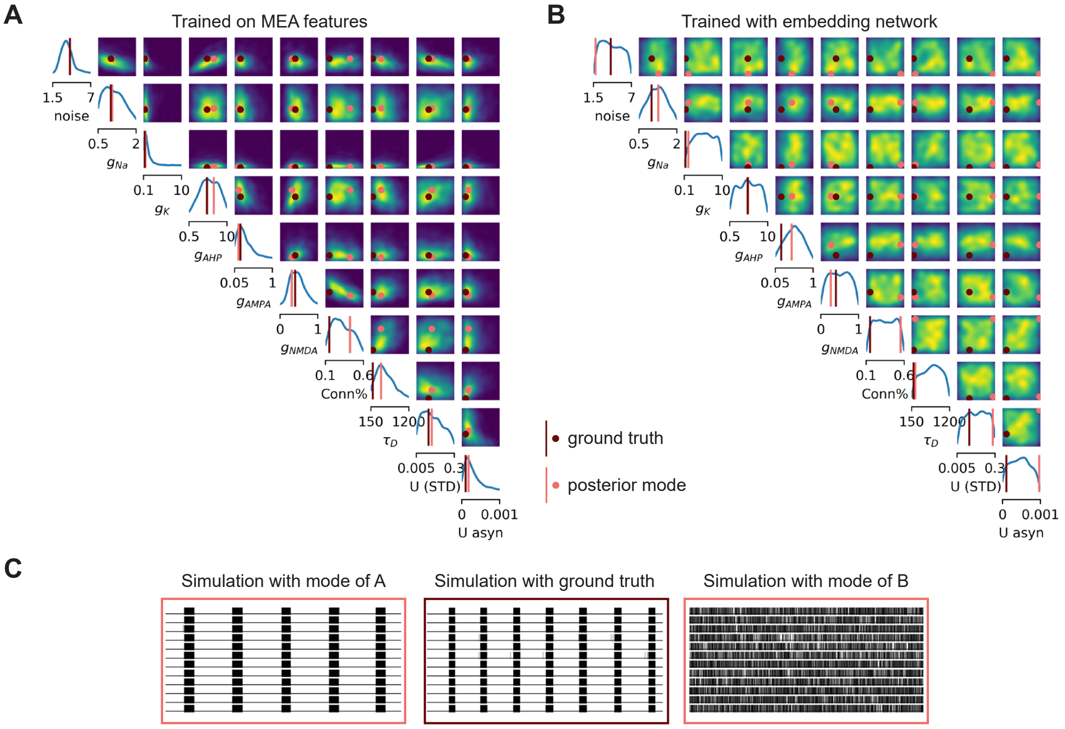

Figure S5: **Comparison of posterior inference using hand-crafted versus data-driven summary features. A)** Posterior distribution inferred from synthetic data using a neural density estimator (NDE) trained on hand-crafted MEA features. **B)** Posterior distribution inferred from the same synthetic data using an NDE trained jointly with a 2D-convolutional embedding network, applied to multi-unit firing rate data. The resulting marginals are noticeably wider and less informative than in A. **C)** Raster plots showing 1 minute of simulations with the ground-truth parameters and the modes of the posterior distributions depicted in panel A and B. While the simulation resulting from the MEA-features approach resembles the bursting behavior of the synthetic data, the simulation with the embedding-network approach shows little similarity to either. This result illustrates a negative finding: despite efforts to develop and train a suitable custom embedding network, parameter recovery was less accurate than with expert-defined features. The average parameter recovery error with hand-crafted MEA features was 0.0728, compared to 0.1670 with the embedding network.

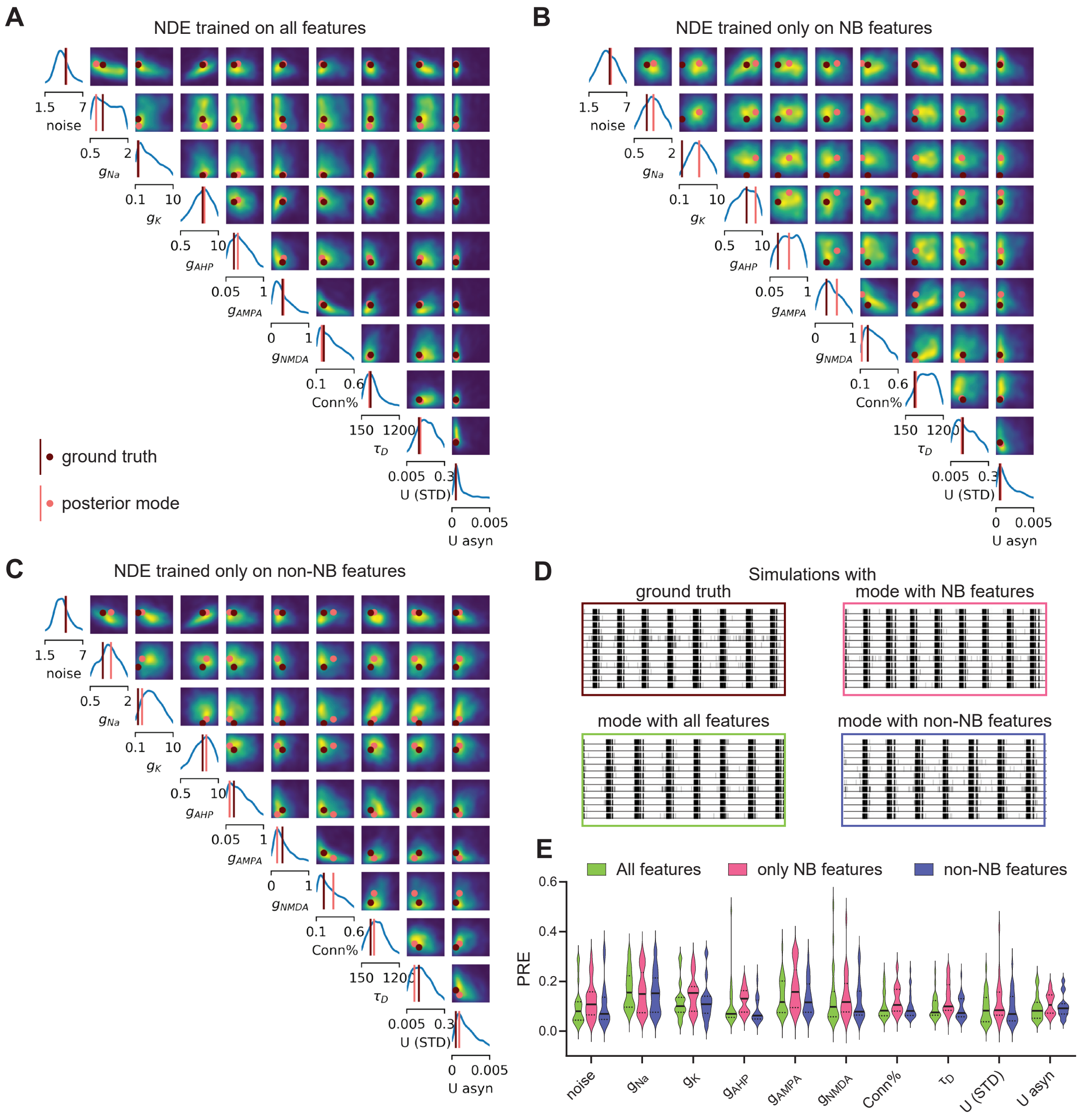

Figure S6: **Effect of training the NDE with different subsets of MEA features on PRE. A-C** Example posterior distributions inferred from synthetic data with the NDE trained on **A)** All 15 MEA features; **B)** Only the 5 Network Burst (NB) related features; and **C)** All features except the 5 NB-related features. **D)** Simulation used as input (ground truth), and simulations with the modes of the posterior distributions shown in A-C. **E)** Violin plots of Parameter Recover Error (PRE) values of 25 evaluations with synthetic data.

References

1. Tolley, N., Rodrigues, P. L., Gramfort, A. & Jones, S. R. Methods and considerations for estimating parameters in biophysically detailed neural models with simulation based inference. *PLOS Computational Biology* **20,** e1011108 (2024).
2. Schmitt, M., Bürkner, P.-C., Köthe, U. & Radev, S. T. Detecting Model Misspecification in Amortized Bayesian Inference with Neural Networks: An Extended Investigation (2024).
3. Ardizzone, L. *et al.* Guided Image Generation with Conditional Invertible Neural Networks (2019).
