# Supplementary material for: Automated inference of disease mechanisms in patient-hiPSC-derived neuronal networks
Source: Commun Biol. 2025 May 20;8:768. doi: 10.1038/s42003-025-08209-2 (PMC12092834; doi:10.1038/s42003-025-08209-2)
Supplement: Supplementary file 3 — Description of Additional Supplementary Files [file 42003_2025_8209_MOESM3_ESM.pdf]

Description of Additional Supplementary Files

File Name: Supplementary Data 1

Description: The source data behind the graphs in the paper, accompanied by all statistical tests and resulting p-values.
